# Research Regarding Different Types of Headlights on Selected Passenger Vehicles when Using Sensor-Related Equipment

**DOI:** 10.3390/s23041978

**Published:** 2023-02-10

**Authors:** Jan Vrabel, Ondrej Stopka, Jozef Palo, Maria Stopkova, Paweł Droździel, Martin Michalsky

**Affiliations:** 1 Department of Road and Urban Transport, Faculty of Operation and Economics of Transport and Communications, University of Zilina, Univerzitná 8215/1, 010 26 Žilina, Slovakia; 2 Department of Transport and Logistics, Faculty of Technology, Institute of Technology and Business in České Budějovice, Okružní 517/10, 370 01 České Budějovice, Czech Republic; 3 Department of Sustainable Transport and Powertrains, Faculty of Mechanical Engineering, Lublin University of Technology, 20-618 Lublin, Poland

**Keywords:** traffic safety, headlight, range of visibility, light cone, dazzlement, sensor-related equipment

## Abstract

Statistical surveys show that the majority of traffic accidents occur due to low visibility, highlighting the need to delve into innovative car lighting technologies. A car driver must not only be able to see but also to be seen. The issue of headlight illumination is vital, especially during the dark hours of the night. Therefore, the focus of this article is determining the range of visibility of dipped (low-beam) headlights under specific experimental conditions. We also designed a methodical guideline aimed at identifying the distance at which dipped headlights illuminate the road while a vehicle is in motion. Research conducted on various classes of road confirmed that the Hyundai i40 is best used on higher-class roads, while the Dacia Sandero is better used on lower-class roads due to the shape and spreading out of its light cone. Furthermore, the pros and cons of the distribution of light cones on several classes of road are presented. Sensor-related equipment was also used to investigate light beam afterglow. In particular, an LX-1108 light meter was applied to determine the obstacle illumination intensity, the properties of which enable recording of low lighting values, and a DJI Mavic AIR 2 unmanned aerial vehicle (UAV; drone) was utilized to record the data related to the location of the examined vehicle, as well as light afterglow at night; relevant data evaluation was carried out using Inkscape software.

## 1. Introduction

Car headlights have recently undergone incremental improvements. The current trend is to equip headlights with properties similar to those of daylight, thereby creating the most favorable conditions possible for drivers [1]. In general, illumination of the vehicle is used mainly to ensure its proper detection on all classes of road, parking spaces and other traffic-related places, playing a crucial role in traffic management and control [2]. Rapid nighttime detection and recognition of vehicles in motion represent one of the most significant and demanding functions of various traffic and transport systems (e.g., standard vehicle equipment, passive and active safety systems of vehicles, intelligent transportation systems, etc.). In recent years, the number of vehicles (although with better safety systems installed) on roads has considerably increased, which has resulted in a stagnant quantity of accidents [3].

Under dark conditions, given the lack of illumination, almost the entire vehicle remains nearly invisible to other traffic participants; therefore, vehicle visibility in such an environment represents a serious issue for road safety [4]. Hence, during nighttime driving, the shape, visibility, condition and light intensity of vehicle headlights in the dark represent a major issue and challenge [5]. 

On the other hand, drivers often counterproductively install and turn on high-intensity headlights, which can inconvenience oncoming drivers (i.e., drivers in the opposite direction) by generating excessive glare, dazzling and sometimes even temporary blindness of the drivers. Therefore, most traffic accidents occur in dark conditions, making this an issue of key importance [6,7]. 

In this manuscript, we analyze two types of headlights—halogen and xenon—and explore the connection between the light source of the headlight and the spatial distribution of the light cone on the road. Thus, the aim of this manuscript is primarily to deal with the field of road traffic safety from the standpoint of its most vulnerable participants. Given the extensiveness of the topic addressed and the quantity of aspects that affect road safety [8], an analysis of contemporary literature in terms of visibility of pedestrians, cyclists and elements on the road was carried out. Furthermore, an overview of literature focusing on road traffic accidents aimed at the most vulnerable participants was processed [9]. With regard to the key objective of the manuscript, last but not least, literature sources associated with road traffic safety per se, traffic markings and safety systems in vehicles were also studied. 

The novelty of the article lies in the fact that it includes a draft investigation procedure according to the elaborated methodology to determine the conditions for measuring the amount of light emitted by headlights of selected passenger cars, along with a description of measuring instruments utilized.

The remained of this manuscript is organized as follows. First, a comprehensive literature review is elaborated, encompassing a series of topic-related works, followed by the methodological section, wherein the applied procedure, along with the relevant methods, is summarized. Thereafter, the most important parts of the conducted research are incorporated, including the investigation itself, and the partial outcomes are discussed in detail. Then, the crucial findings and accompanying discussion are presented. In these sections, we present experimentally measured values listed in the form of tables, photographs and figures to model the distribution (spreading out) of light cones on different classes of road. Last but not least, related conclusions derived from this research study are outlined. Specifically, the aim of this manuscript is to evaluate and compare the results of experimental measurements and present particular recommendations for further research on the addressed topic.

## 2. Literature Review

The subject of nighttime visibility of various means of transport has been discussed in a wide array of scientific publications [10,11,12,13,14]. For instance, Neale et al. described the impact of the moon and its brightness on the illumination of pedestrians on roads [10]. The authors examined the intensity of pedestrian illumination with respect to the position of the moon in its individual phases. Rosey et al. addressed the visibility of distinct obstacles in road traffic [11]. They investigated several dozen drivers driving on two-lane rural roads and highways in a simulator and tested various visibility conditions. The results of their research revealed differences in the behavior of drivers on such classes of road with a visibility of 30 m. According to Abdur et al., poor sensory visibility, as well as poor bicycle visibility, are key factors that correlate with bicycle and vehicle accidents [12]. Their study resulted in recommendations for cyclists regarding additional equipment and technical devices for bicycle recognition on roads in order to improve their visibility to drivers of oncoming vehicles.

In [13], the authors investigated technology to assist drivers in detecting objects under conditions of reduced visibility by implementing projection light beams (cones) in vehicle headlights. Their research compared several sets of duplicate testing vehicles (i.e., identical manufacturer model and model year). According to the authors, even with the same vehicles, headlights from the same manufacturer in vehicles of the same model and model year have different headlight configurations and options, which affect the light beam of the vehicle headlights. According to the results of the study elaborated by Black et al. [14], cycling at night is dangerous, a collision with a cyclist from behind represents the main cause of death during night riding. In their study, the authors measured a series of safety features that can be used by cyclists to increase visibility. 

With respect to the topic of traffic accidents and crashes in relation to vehicle illumination, several works were reviewed [15,16,17,18,19]. In [15], the authors compared the development of fatal traffic accidents in the USA and observe the linear regression of such accidents with respect to multiple related factors, presenting a general conclusion and recommendation that advanced lighting of roads and proper shape, configuration and type of vehicle headlights be implemented to improve the visibility of pedestrians under dark conditions. According to the authors of [16], criteria for the design of highway shapes are mainly based on driving conditions during the day and therefore disregard driving conditions at night. From a safety point of view, the authors focused on possible encounters of vehicles with animals at night, which are typical, e.g., in areas of the USA and Greece, and represent the worst possible scenario under conditions of minimal illumination and reduced visibility. 

Yang et al. investigated the relationship between lighting photometric measures and the risk of crashing into an obstacle in road traffic during under dark conditions on individual sections of roadways examined in the USA [17]. On the other hand, in 2010, research was conducted with the aim of determining whether there is an interconnection between vehicles deployed on US roads equipped with daytime running lights (DRL) and the occurrence of traffic accidents [18]. Measurements were collected for vehicles registered in Minnesota equipped with DRL and compared to the total number of registered vehicles. A study with a similar intention was carried out by Sullivan and Flannagan (2007) [19], who defined the impact of light intensity for three crash-related scenarios in relation to three adaptive headlight approaches, i.e., highway lighting, curve lighting and cornering lighting. The risk related to the intensity of lighting was specified for the obtained individual outcomes by calculating the value of daylight-saving time transitions in order to identify the dark (light) interval ratio of risk.

A number of literature sources have been published concerning the issue of traffic safety in association with vehicle lighting [20,21,22,23,24,25]. In [20], the authors dealt with the findings of a traffic survey conducted on Nigerian roads. Based on research findings, they ascertained several shortcomings and recommended introducing enhancement measures and strategies that can help to increase road safety, improve traffic planning and mitigate emerging problems in the field of road transport in Nigeria. According to Reagan et al., an increase in road traffic safety can be achieved through the use of automatic main (high) beam switching systems [21]. The authors conducted research on this particular subject, reporting outcomes that were much worse than their expectations. In [22], the authors focused on monitoring and comparing different headlight source technologies in low-beam mode in combination with and without street lighting in order to evaluate the visibility of other road users in specific traffic situations. In [23], the authors investigated traffic safety in terms of intersection illumination by street lighting and vehicle headlights and the impact of illumination on the frequency of crashes and the occurrence of property damage.

Jawi et al. [24] investigated road traffic safety in relation to the traffic accident rate. Specifically, the authors focused on the accident rate of motorcycles and found that dazzling of drivers by the main beams used by other vehicles moving in the opposite direction is the main reason for the occurrence of traffic accidents. Their research dealt with the distance traveled by vehicles during the switch between main and low-beam headlights. Analogously, the authors of [25] discussed the topic of the traffic accident rate in relation to the use of headlights under dark conditions. In experiments, they applied computer simulation to detect and compare the distance and reaction time associated with multiple headlight types. 

In several literature sources [26,27,28,29,30], authors investigated horizontal road markings from different points of view. For instance, the authors of [26] highlighted the issue of headlight reflection with respect to the use of retroreflective materials. Based on the results of field experiments, the authors of [27] highlighted that the choice of premium glass beads significantly extends the durability of renewed road markings, as calculated in relation to material savings, diminishing the emissions of volatile organic compounds, in addition to reducing financial costs. Similar studies were elaborated by the authors of [28,29], who targeted visibility under wet nighttime conditions, in particular with respect to traffic markings in urban agglomerations. In [30], Schnell and Zwahlen investigated the visibility of pavement markings for drivers in urban areas using a retroreflective approach. The authors also executed a series of examinations in which the longitudinal focus of the eyes on straight roads illuminated by low beam headlights was scanned.

With respect to road lighting, many authors have investigated the implementation of adaptive headlights or other advanced or smart technological solutions installed in up-to-date vehicles, such as various technologies of advanced control systems [31,32,33,34,35]. In [31], Chen and Chiu described an adaptive headlight control system (AHS) and suggested an option to retrofit passenger cars, trucks and other vehicles. They stated that although such systems improve lighting angles and the visibility of road obstacles, their installation in vehicles is associated with increased costs. Therefore, they recommended that such systems be introduced in high-end vehicles. The authors of [32] offered novel insight into road lighting, focusing on the development of an efficient image processing algorithm that ensures a sufficient level of lighting intensity for drivers, simultaneously ensuring a minimal level of intensity for drivers traveling in the opposite direction.

Additional studies [33,34] were conducted on the basis of a similar principle, in which the authors experimentally minimized oncoming driver dazzlement by reducing the brightness of the vehicle headlight main beam. Reductions in brightness and driver dazzlement were ensured by changing the voltage supplied to the headlights based on vehicle speed data and an embedded programming language. The effect of the introduction of an AHS on pedestrian deaths in the dark was addressed by Subramanian et al. [35], who investigated how pedestrians respond to various AHS technologies. They conducted research on an experimental sample of 106 adults and concluded that the AHS approach can have a considerable positive impact on safety when pedestrians cross the road.

In addition, Beddar et al. outlined the use of LED technology in the automotive and aerospace industries, which can contribute to energy reduction in these sectors and improve the visibility of obstacles at long distances without disturbing attention or increasing the discomfort of other road traffic users [36]. On the other hand, the authors of [37] described of how adequate bicycle illumination under dark conditions can address deficiencies in public transport relating to cyclist injuries. However, using such illumination, a cyclist may be dazzled or an accident with a cyclist may occur. As a result, proper bicycle illumination and dazzlement prevention can help to reduce the quantity of cyclist injuries at night. Finally, Villa et al. reported a case study with the main objective of examining a nighttime environment on roads with an oncoming motorcycle and conducted two experiments concerning motorcycle motion recognition with a sample of 33 participants [38]. The obtained results imply that motion stimuli at night are more realistic and authentic with an HDR display, which may be useful to ensure the necessary realistic recognition of difficult-to-see contrasts.

Table 1 summarizes the results of our literature review.

## 3. Materials and Methods

### 3.1. Proposed Methodology for Measuring the Range of Visibility

Based on the findings of the research studies presented above in the literature review, we came to the conclusion that the optimal period to carry out investigations regarding road and pedestrian dazzling is that in which the moon is in a new phase, as the brightness of the moon (dazzlement) cannot affect measured data during this period. In addition, for such investigations, pedestrians should not be wearing reflective elements, and the investigation should be conducted on a night with a clear sky. Furthermore, the road under examination should not contain any retroreflective elements or horizontal/vertical traffic signs.

The aim of the suggested methodology is to identify the distance for which dipped headlights illuminate the road when a vehicle is in motion. The following conditions apply:

1. The segment of road used for the measurements must be horizontal or have a steady incline and must not contain any obstacles or other sources of light that can impact the measured values;
2. The test must take place at nighttime in weather conditions without atmospheric precipitation;
3. The headlights must be set in the correct position, and the cover glass must be cleared of dirt;
4. The person illuminated by the headlights must not wear black, white or reflective clothing. A person wearing white clothing would reflect the light, whereas in contrast, black clothing would absorb the light. As reflective clothing would also distort the visibility of the figure, it was decided to use gray clothing.

The determined procedure is as follows:

1. The vehicle is moved into a marked position on the selected segment of road. The position is approximately in the middle of the road. Before moving the vehicle to the marked position, the incline of the road is measured using a laser gauge and a suitable GIS application.
2. The height between the headlights and the road surface and the distance between the two headlights are measured using a tape measure. When the headlights are switched on, the unlit area in front of the vehicle is marked using spray.
3. The intensity of illumination is measured at two points: 54 cm above the road surface, i.e., knee height, at the road surface level, i.e., ankle height, which is approximately 10 cm above the road surface. As the illuminated person walks away from the light source, the observer determines at what point the knees become illuminated. This point is subsequently marked with spray. Observations are conducted step by step from the left side of the road to the right side of the road in half-meter intervals. The procedure is the same for ankle-height observations, with the positions marked with a different color of spray.
4. The term ‘range of visibility’ is used to describe the distance illuminated by dipped headlights in one vehicle lane. With the dipped headlights switched on, the illuminated person moves away from the light source step by step while the observer checks the visibility of the shadow cast by the pedestrian’s footwear. The distance at which the shadow disappears determines the range of visibility. Once again, the test takes place in the dark, with the pedestrian crossing the road from left to right in half-meter intervals. The points are marked using spray of a third color.
5. The nighttime test concludes by marking the light cones from a bird’s eye point of view using a drone. The same is done for the two illuminated points inside the light cone at the identified distances from the vehicle.
6. The second measurement step takes place during daylight hours. The distances between the spray-marked points on the road from the vehicle are determined using a drone.

### 3.2. Sensor-Related Equipment

Several accessories were used to examine light beam afterglow, which are related to the issue of sensors to varying extents.

First, an LX-1108 light meter was used to determine the obstacle lighting intensity, the properties of which enable measurement even low lighting values (see general specifications in Table 2) [39].

In order to record the light afterglow length at night, i.e., the place where the light beam was located at knee or ankle level, a specific spot was marked on the road with a colored spray. Likewise, the vehicle position was marked to ensure comparability of measurements. Subsequently, a photo of the location under investigation was taken using a drone. A DJI Mavic AIR 2 unmanned aerial vehicle (hereafter referred to as drone) was used to record the relevant data (see the specifications of the DJI Mavic AIR 2 Drone in Table 3) [40], and data evaluation was carried out using Inkscape software.

The headlight afterglow was examined based on a modified original measurement methodology. The benefit of this research lies in the incorporation of a drone to locate the exact sensory position of the vehicle and determine its light beam intensity. Based on the data acquired using a uniform methodology, it is possible to compare the measurement findings. The data obtained in this way can contribute to the investigation of traffic accidents that occur at night. Using this approach, it is possible to carry out examinations of all types of headlights from different vehicle manufacturers, thereby assisting in clarifying traffic accident questions, for example, whether the driver could have prevented a collision with a pedestrian, an animal or another participant in the traffic accident. Based on the obtained data, a decision can be made as to whether or not the driver could detect an obstacle in road traffic at a given vehicle velocity and therefore prevent the vehicle from colliding with it.

### 3.3. Description of the Investigation and Partial Results

**Figure 1:**
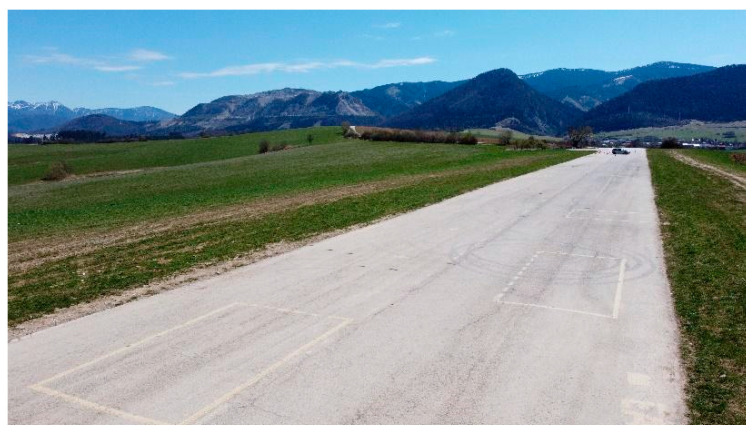
Runway of Rosina Airport.

Investigations involving observations on two nights and during two days took place from 27 April to 29 April 2021 on the runway (hereafter referred to as “road”) of Rosina Airport in the vicinity of Žilina. The sky was cloudy throughout the nighttime tests, so moonlight had no impact on the results. The illumination intensity of the cloudy night sky without other illumination was 0.0001 1×. The daytime tests took place when the sky was partly cloudy without atmospheric precipitation. The lighting conditions were the same throughout. A suitable segment of road was found, i.e., one with the lowest longitudinal and transverse incline and no (other/artificial) light sources (see Figure 1).

**Figure 2:**
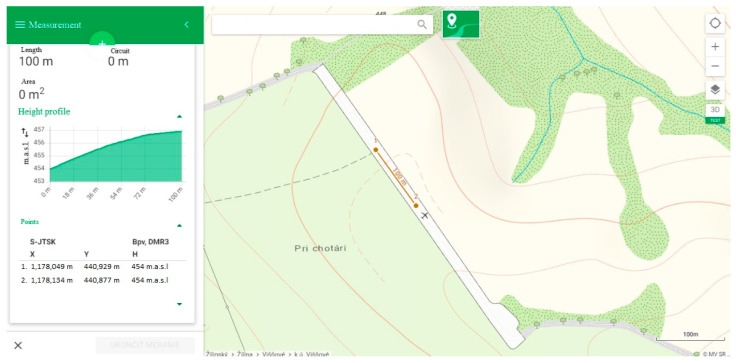
Incline of the measured segment of road according to ZBGIS.

The ZBGIS portal—the Internet map client and web application operated by the national cadastral office of the Slovak Republic for displaying, searching and analyzing spatial data of the real estate cadaster—was used to determine the angle of the incline of the selected segment of road. The difference in elevation was 3 m over a distance of 100 m, i.e., 3% (see Figure 2). The tests took place in the rising direction [41].

**Figure 3:**
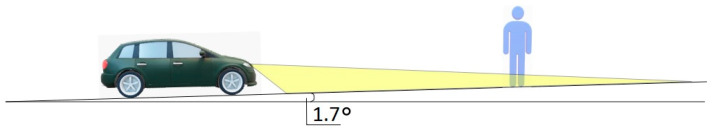
Incline of the road against an obstacle.

We verified the figures from the ZBGIS portal using a 2 meter level and a BOSCH GLM 500 digital slant gauge [41]. The angle was determined to be 1.7°, which is an equivalent of an incline of 2.9665% on the 100 m segment (see Figure 3). It should be noted that the incline of the road has no effect on the range of visibility or illuminated obstacles because the vehicle and the road surface are at the same angle.

The tests were conducted using two vehicles with different types of headlights. Technical data of the vehicles using both types of headlights are listed in Table 4 and Table 5.

**Figure 4:**
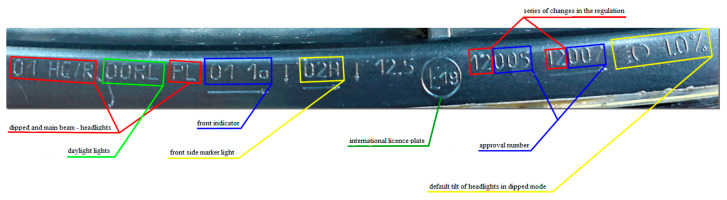
Headlight approval plate of the Dacia Sandero. Code E19—Romanian identification number; HC/R—halogen headlight, Class B headlight; PL—headlight cover glass is made of plastic. According to the imprinted marks, the headlights are meant for left-hand-drive vehicles.

The headlight approval plate of the investigated Dacia Sandero is presented in Figure 4.

The investigated headlights complied with the requirements of regulations governing vehicles that fall in the M1 category, namely Regulation No. 20 EHK OSN on the adoption of uniform technical prescriptions for wheeled vehicles, which requires asymmetric headlights in dipped or main beam mode (or both) equipped with filament light bulbs (H4), as well as Regulation No. 99 EHK OSN on the adoption of uniform technical prescriptions for light sources with a gas discharge lamp used in approved lights for wheeled vehicles [42].

The experiment followed the devised methodology. Before any measurements were taken, dust and dirt were removed from the headlights to prevent light scattering. Nighttime measurements were conducted first, followed by daytime measurements. In both cases, the experiments were carried out under drone surveillance (drone type: DJI Mavic Mini).

The first part of the nighttime tests focused on determining the range of visibility at two heights: knee height (app. 54 cm above the road surface level) and ankle height (app. 10 cm above the road surface level). The second part involved identifying the maximum range of visibility of the headlights by detecting the borderline between the light cone and the unlit part of the road. The process always proceeded according to the adopted methodology, i.e., from the left side of the road, where we marked the first test point with a colored spray. The illuminated person crossed to the right side of the road, spray-marking the range of visibility every half-meter (represented by twelve points on the road surface). The marked points formed the basis for the subsequent modeling of the light cone. The distances between the measured points and the vehicle were noted in meters during the daytime observations, with a drone monitoring the situation throughout. The points were marked in different colors to make the test clearer. To indicate which vehicle was involved in each experiment, the Dacia Sandero bore a line, while the Hyundai i40 bore a cross.

**Figure 5:**
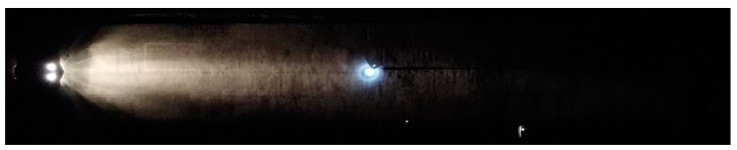
The illuminated person at a distance of 31.85 m from the Dacia Sandero.

**Figure 6:**
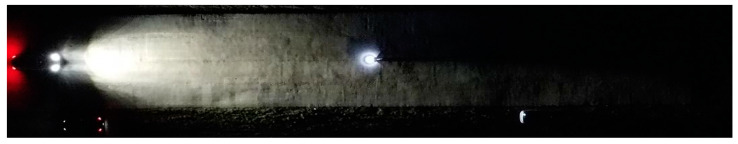
The illuminated person at a distance of 64.70 m from the Dacia Sandero.

**Figure 7:**
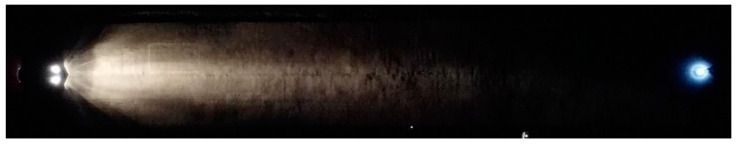
The illuminated person at a distance of 31.85 m from the Hyundai i40.

**Figure 8:**
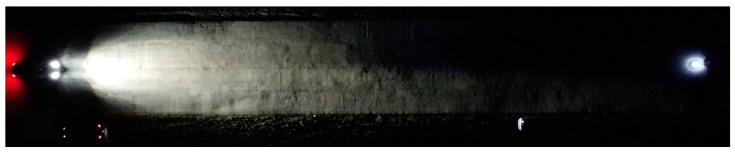
The illuminated person at a distance of 64.70 m from the Hyundai i40.

During the nighttime experiments, a person carrying an LED torch entered the light cone, first at a distance of 31.85 m and then 64.70 m from the vehicle. The measurement demonstrated the distribution of the light cone against the illuminated obstacle in front of the vehicle. Figure 5, Figure 6, Figure 7 and Figure 8 illustrate the situation in terms of illumination for both vehicles.

The nighttime experiment revealed that the headlights of the Hyundai i40 form a light cone of a higher intensity than of the Dacia Sandero. The results show that the higher intensity of the headlights of the Hyundai i40 more effectively illuminate the environment in front of the vehicle.

**Figure 9:**
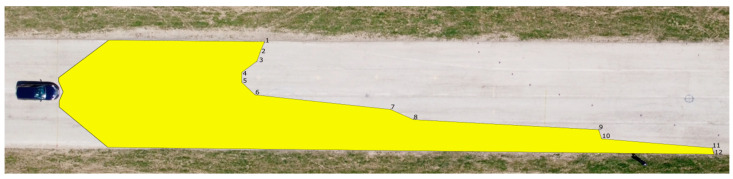
Range of visibility at knee height—Dacia Sandero.

**Figure 10:**
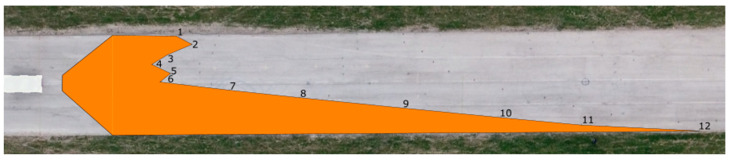
Range of visibility at knee height—Hyundai i40.

The determined values of the distances from the vehicles for the 12 measured points in relation to the range of visibility at knee height are listed in Table 6. A drone took pictures of the twelve designated points during the daytime tests. The photographs were subsequently exported into Inkscape graphic editing software. The distribution of the light cones, including the twelve measured points, is depicted in Figure 9 and Figure 10.

The range of visibility at knee height (54 cm above the road surface) of the dipped headlights of the Dacia Sandero was determined to be 67.22 m, while that of the Hyundai i40 was 78.11 m. The light cone produced by the Dacia Sandero lit up a larger area in front of the vehicle, while that of the Hyundai i40 reached further on the right side of the road.

**Figure 11:**
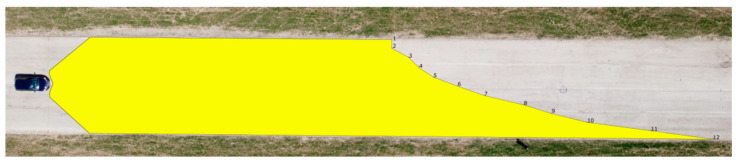
Range of visibility at ankle height—Dacia Sandero.

**Figure 12:**
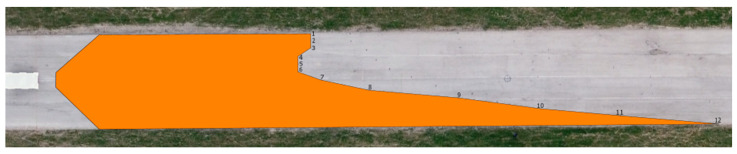
Range of visibility at ankle height—Hyundai i40.

We applied the same procedure to measure the range of visibility at ankle height. The results are presented in Table 7 and Figure 11 and Figure 12.

The range of visibility at ankle height (10 cm above the road surface) of the dipped headlights of the Dacia Sandero was determined to be 83.32 m, while that of the Hyundai i40 was 93.44 m. The graphic depiction of the light cones suggests that the Hyundai i40 illuminates the road area asymmetrically, which is imperative to prevent dazzling of oncoming vehicles, whereas this is not as much the case for the Dacia Sandero. 

**Figure 13:**
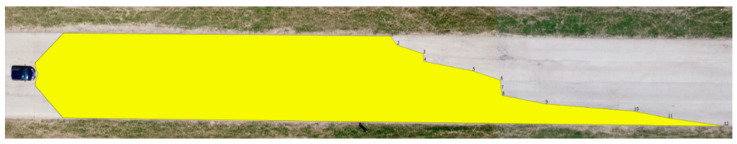
Maximum range of visibility—Dacia Sandero.

**Figure 14:**
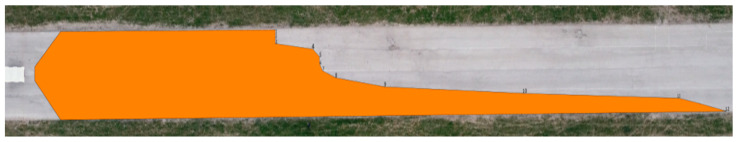
Maximum range of visibility—Hyundai i40.

The final test involved determining the maximum range of visibility on the basis of the borderline between the light cone and unlit part of the road. The outcomes were processed in the same way as the measurements for the range of visibility at knee height and ankle height. The results are presented in Table 8 and Figure 13 and Figure 14.

The measurements suggest that the greater the distance from the vehicle, the lower the illumination intensity at the marked points. With dipped headlights, the maximum range of visibility for the Dacia Sandero was determined to be 123.75 m, while that of the Hyundai i40 was 160.56 m.

## 4. Results and Discussion

The figures reveal that compared to the Dacia Sandero, the Hyundai i40 provides greater visibility on the right side of the road but poorer visibility on the left side of the road. However, a driver of a Dacia Sandero would see an obstacle on the left of the road from a greater distance than a person driving a Hyundai i40. Between the two vehicles, the situation on the right side of the road is completely the opposite. The Dacia Sandero’s light cone covers a much larger area of the road, thereby dazzling oncoming vehicles, as well as those in front. The pictures show that the light cone of the headlights of the Hyundai i40 creates a space that mitigates such dazzling because of the illumination intensity and the homogeneity of the light distribution, which ensures greater safety. The xenon headlights of the Hyundai i40 are brighter than the halogen headlights of the Dacia Sandero, and their color temperature is closer to that of daylight [43].

**Figure 15:**
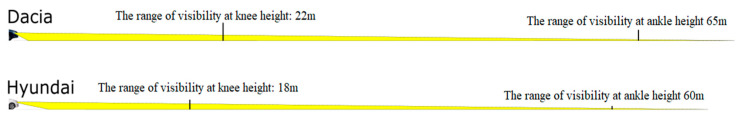
Comparison of theoretical values of the range of visibility at knee height and ankle height.

According to the manufacturers’ data, the headlights of the Dacia Sandero should illuminate the road up to a distance of 75 m, whereas those of the Hyundai i40 should illuminate the road up to 70 m (both in dipped mode). The theoretically determined values of the range of visibility at knee height and ankle height are compared in Figure 15.

The practical experiment revealed that the real values for the range of visibility changes when crossing from the left to the right side of the road. The mean value of the acquired data is not statistically significant, as the light cones show asymmetric distribution on the road. The left side indicates a lower real range of visibility than the right side (to prevent dazzling of oncoming vehicles). The theoretical values of the range of visibility at knee height correlate with the data achieved by measuring the symmetrical axis of the vehicles. The values obtained from testing the range of visibility at ankle height correspond with the values for the range of visibility gathered on the right side of the road. 

However, it is possible that several mistakes were made during the observations. The accuracy of the data could be brought into question if the angle of the headlights is judged to have been incorrectly adjusted or if the cover glass was dirty. Both vehicles had their headlights fixed according to the State Technical Inspection. Other mistakes could have been made when measuring the distances between the individual points and vehicles using a tape measure. Another possible contributing factor is the visual abilities of the observer watching the illuminated person. Eyesight sensitivity has a direct impact on how someone perceives the contrast between light and dark. The values determined for the range of visibility at knee height and ankle height are therefore dependent on the observer’s own visual sensitivity, as well as on the ability of the illuminated person to reflect the light from the headlights.

In the subsequent paragraphs, we compare the spatial (modeled) distribution of the light cones created by the headlights of the vehicles on different classes of road.

**Figure 16:**
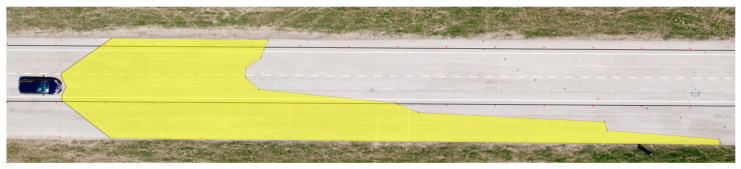
C 6.5 (III) class road—knee-height illumination—Dacia Sandero.

**Figure 17:**
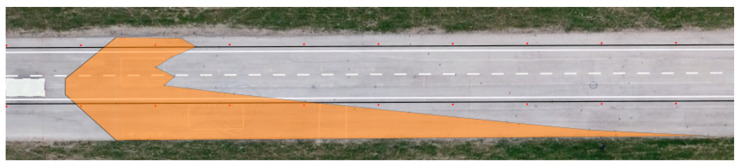
C 6.5 (III) class road—knee-height illumination—Hyundai i40.

**Figure 18:**
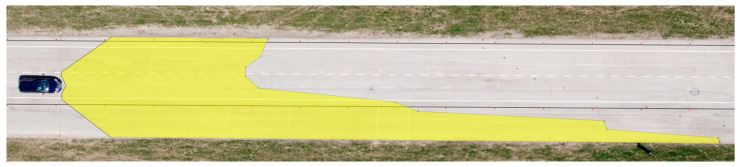
C 7.5 (II, III) class road—knee-height illumination—Dacia Sandero.

**Figure 19:**
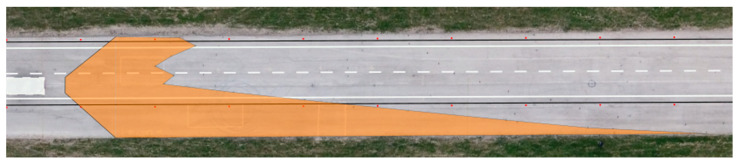
C 7.5 (II, III) class road—knee-height illumination—Hyundai i40.

**Figure 20:**
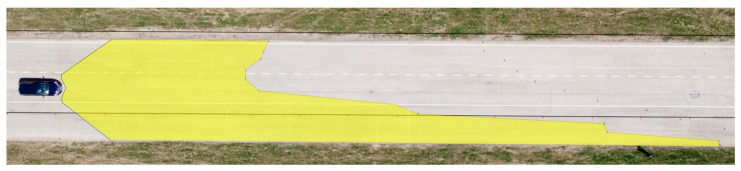
C 9.5 (I, II, III) class road—knee-height illumination—Dacia Sandero.

**Figure 21:**
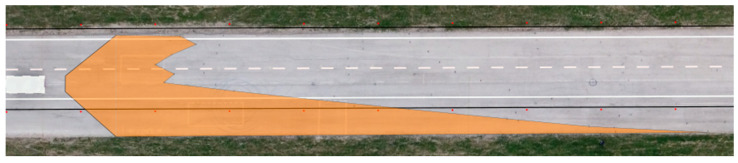
C 9.5 (I, II, III) class road—knee-height illumination—Hyundai i40.

**Figure 22:**
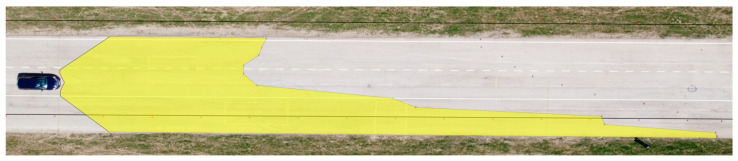
C 11.5 (I) class road—knee-height illumination—Dacia Sandero.

**Figure 23:**
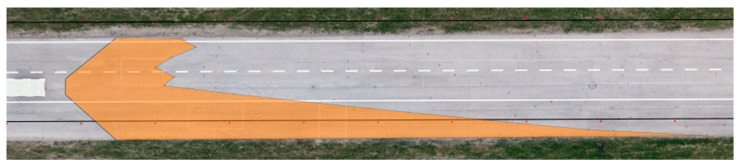
C 11.5 (I) class road—knee-height illumination—Hyundai i40.

**Figure 24:**
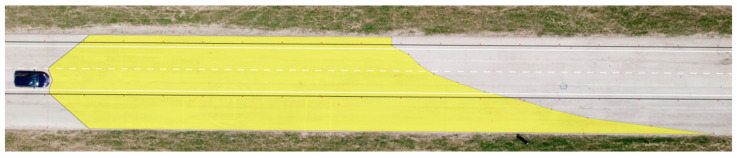
C 6.5 (III) class road—ankle-height illumination—Dacia Sandero.

**Figure 25:**
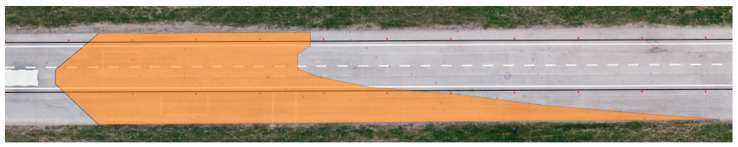
C 6.5 (III) class road—ankle-height illumination—Hyundai i40.

**Figure 26:**
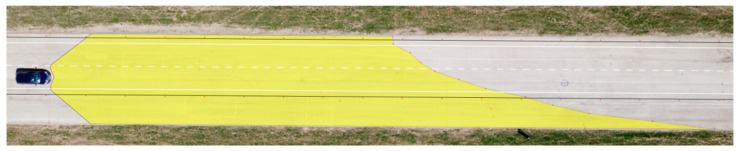
C 7.5 (II, III) class road—ankle-height illumination—Dacia Sandero.

**Figure 27:**
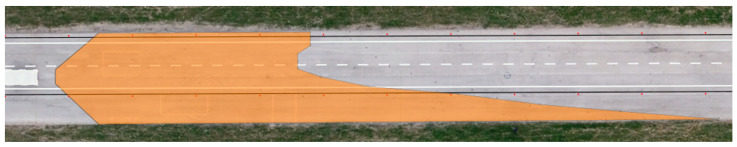
C 7.5 (II, III) class road—ankle-height illumination—Hyundai i40.

**Figure 28:**
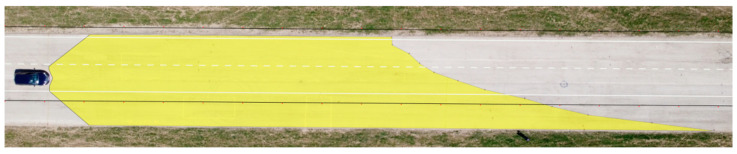
C 9.5 (I, II, III) class road—ankle-height illumination—Dacia Sandero.

**Figure 29:**
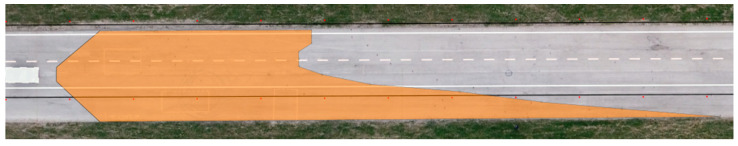
C 9.5 class road (I, II, III)—ankle-height illumination—Hyundai i40.

**Figure 30:**
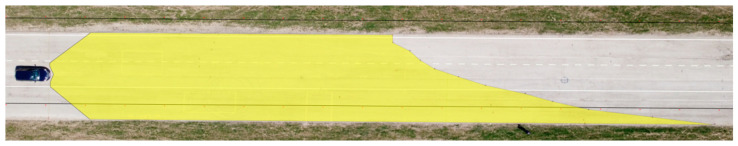
C 11.5 (I) class road—ankle-height illumination—Dacia Sandero.

**Figure 31:**
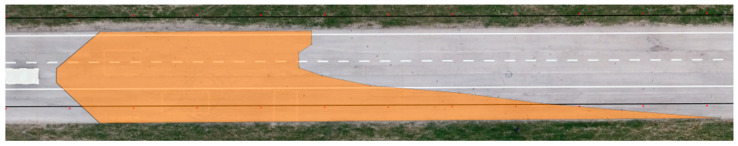
C 11.5 (I) class road—ankle-height illumination—Hyundai i40.

The widths of individual structural elements of roads in non-urban areas are stipulated in STN 73 6101, ‘Design of Highways and Motorways’ [44]. The widths of traffic lanes and hard shoulders were entered into the Inkscape program. The acquired data were subsequently imported into the Inkscape graphic editor. Figure 16, Figure 17, Figure 18, Figure 19, Figure 20, Figure 21, Figure 22, Figure 23, Figure 24, Figure 25, Figure 26, Figure 27, Figure 28, Figure 29, Figure 30 and Figure 31 depict the modeled spatial distribution of the light cones on the identified class of road. The blue circle indicates a pedestrian walking along the side of the road. The figures show the pedestrian at the furthest point from the vehicle at which the driver is able to spot the specified part of the pedestrian’s body. 

As shown in Figure 16, Figure 17, Figure 18, Figure 19, Figure 20, Figure 21, Figure 22, Figure 23, Figure 24, Figure 25, Figure 26, Figure 27, Figure 28, Figure 29, Figure 30 and Figure 31, the Dacia Sandero, with its light cone, covers a larger area of road in front of the vehicle than the Hyundai i40, although its light cone is spread out in such a way that it mainly illuminates the peripheral parts beyond the edge of the road (i.e., outside the main traffic) and does not dazzle the drivers of oncoming vehicles [45].

Table 9 compares the range of visibility of a pedestrian at knee height walking on the side of the selected classes of road for the two vehicles.

Table 9 reveals that the Dacia Sandero offers greater visibility of a pedestrian at knee height when they are walking on the side of a road, while the Hyundai i40 provides wider coverage on C 9.5 class roads due to the more suitable shape of the light cone. Although the Dacia Sandero provided a larger range of visibility in most cases, the intensity of the light was significantly lower than that of the Hyundai i40. The headlights of the Dacia Sandero, which are positioned 5 cm higher than the headlights of the Hyundai i40, also had an impact on the achieved values. 

Table 10 compares the range of visibility of a pedestrian at ankle height walking on the side of the selected classes of road for the two vehicles.

Table 10 proves that the Dacia Sandero offers greater visibility of a pedestrian at ankle height when they are walking on the side of a road, whereas the Hyundai i40 provides wider coverage on C 11.5 class roads due to the higher intensity of the light source. The headlights of the Dacia Sandero could not meet the requirements for the illumination intensity at this specific point, i.e., the illumination decreased in intensity with distance. The headlights of the Dacia Sandero, which are positioned 5 cm higher than the headlights of the Hyundai i40, also had an impact on the achieved values. 

As demonstrated by the data presented above, the modeling of the light cones on various classes of road shows that the Hyundai i40 is best used on higher-class roads. On the contrary, the Dacia Sandero is better used on lower-class roads due to the shape and distribution of its light cone.

The primary objective of this research was to design a measurement methodology, with the examined acting as representative samples for the research. The measured data and the data obtained from the footage captured using a drone were processed by a graphic editor in order to acquire a light beam in a form of vector display. As a result of this processing, it was possible to apply such a light beam form to different road alignments, types and classes, in accordance with the technical requirements for the construction of various road categories in the Slovak Republic. This approach can be generally applied to any road alignment and classes in any state, as the light cone obtained in this investigation is sufficiently wide. Findings for different categories, brands and models of vehicle can be achieved by further research following the methodology suggested in this article.

## 5. Conclusions

As previously mentioned, the emphasis of this research was the most vulnerable participants in road traffic. To this end, a comprehensive literature review was conducted in order to take into account all intricacies when considering the range of the subject, as well as the quantity of factors influencing the issue of road safety. Therefore, we reviewed literature on road traffic safety; visibility of pedestrians, cyclists and elements on the road; traffic accidents; traffic markings; and safety systems in vehicles.

With respect to the obtained results, a detailed comparison of halogen and xenon headlights revealed that the xenon headlights of the Hyundai i40 were better suited to the prevailing conditions on dual carriageways and higher-class roads because they emit a brighter light that covers a larger range of visibility than the headlights of the Dacia Sandero. The Dacia Sandero produces less intense light but better illuminates obstacles within a distance of 67 m. A significant change occurs at distances further than 67 m, after which the range of visibility parameters work better for the Hyundai i40. The modeled light cones clearly show that the Dacia Sandero dazzles oncoming vehicles and vehicles in front. This shortcoming is partly the result of the lower illumination intensity. The asymmetrical distribution of the light cone of the Hyundai i40 enables better illumination of the right side of the road, thereby dazzling oncoming vehicles and vehicles in front, less. The comparison of the light cones on different classes of road suggests that the Dacia Sandero is better used on lower-class roads due to the distribution of the light cone.

Based on the analysis carried out in this study and the data obtained from the experiments, we can conclude that the examined dipped (low-beam) headlights are seemingly appropriate to light roads of lower classes, although this conclusion must be verified by further investigations involving vehicles with LED headlights or analog high-definition (AHD) systems, as well as comparisons of research results.

## Figures and Tables

**Table 1:** Summary of the literature review.

Topic	Authors of the Reference	Issue Addressed
Nighttime visibility of various means of transport	Neale et al. (2019)	Impact of the moon and its brightness on illumination of pedestrians on roads.
	Rosey et al. (2017)	Driver behavior in fog and the visibility of distinct obstacles in road traffic.
	Abdur et al. (2021)	Poor sensory visibility and bicycle visibility as the key factors correlating with bicycle and vehicle accidents.
	Funk et al. (2021)	Why is it useful to help drivers to detect object under conditions of reduced visibility by implementing projected vehicle headlight beams?
	Black et al. (2020)	Measurement of safety features used by cyclists in order to increase their visibility.
Traffic accidents and crashes in relation to vehicle illumination	Hu and Cicchino (2018)	The development of fatal traffic accidents in the USA and observation of the linear regression of such accidents with respect to relating factors.
	Papadimitriou and Psarianos (2015)	Possible encounters of vehicles with animals at night, which are typical, e.g., om the areas of the USA and Greece, representing the worst possible scenario under conditions of minimal illumination and reduced visibility.
	Yang et al. (2019)	The relationship between lighting photometric measures and the risk of crashing into an obstacle in road traffic during under dark conditions on US roadways.
	Krajicek and Schears (2010)	Is there an interconnection between vehicles deployed on US roads equipped with daytime running lights and the occurrence of traffic accidents?
	Sullivan and Flannagan (2007)	Impact of light intensity in three crash-related scenarios in relation to three adaptive headlight approaches, i.e., highway lighting, curve lighting and cornering lighting.
Traffic safety in association with vehicle lighting	Salisu and Oyesiku (2020)	Recommendations for enhancement measures and strategies that can help to increase road safety, improve traffic planning and mitigate emerging problems in the field of road transport in Nigeria.
	Reagan et al. (2017)	Increased road traffic safety in terms of the use of automatic main (high) beam-switching systems.
	Baleja et al. (2019)	Monitoring and comparison of headlight source technologies in low-beam mode in combination with and without street lighting to evaluate the visibility of road users.
	Goswamy et al. (2018)	The impact of illumination on the frequency of crashes and the occurrence of property damage in terms of intersection illumination either by street lighting or vehicle headlights.
	Jawi et al. (2020)	The accident rate of motorcycles with respect to dazzling of drivers by the main beams used by other vehicles moving in the opposite direction, which is the main reason for the occurrence of traffic accidents.
	Lee et al. (2014)	Investigation of the traffic accident rate with respect to the use of headlights under dark conditions by applying computer simulation to detect distance and reaction time.
Horizontal road markings in the context of visibility	Shin et al. (2019)	Headlight reflection with respect to the use of retroreflective materials.
	Burghardt et al. (2021)	The choice of premium glass beads as an aspect that significantly extends the durability of renewed road markings.
	Amparano and Morena (2006)	Improved visibility under wet nighttime conditions with respect to traffic markings in urban agglomerations.
	Gibbons et al. (2005)	
	Schnell and Zwahlen (1999)	Pavement markings in urban areas using a retroreflective approach.
Adaptive headlights and other advanced or smart technological solutions in terms of road lighting	Chen and Chiu (2018)	Adaptive headlight control system and the option to retrofit passenger cars, trucks and other vehicles.
	Harindu et al. (2020)	The development of an efficient image processing algorithm that ensures a sufficient level of lighting intensity for drivers.
	Ajay et al. (2019)	Minimization of the dazzlement of oncoming drivers by reducing the brightness of main vehicle headlight beams through the introduction of advanced computing technology.
	Somasundaram et al. (2020)	
	Subramanian et al. (2021)	Adaptive headlight control systems in relation to pedestrian deaths in the dark.
LED technology in the automotive and aerospace industries	Beddar et al. (2020)	Energy reduction and improved obstacle visibility at long distances using LED headlights without disturbing attention or increasing the discomfort of road traffic users.
Adequate bicycle illumination in order to reduce cyclist injuries	Dudziak and Caban (2021)	How can proper bicycle illumination under dark conditions address deficiencies in public transport relating to cyclist injuries?
Improvement of the realism of motion stimuli using HDR displays	Villa et al. (2018)	Are motion stimuli at night more realistic and authentic with high-dynamic-range displays, and might such technology be useful for the necessary realistic recognition of difficult-to-see contrasts?

**Table 2:** General specifications of the LX-1108 light meter.

Measurement and Range	5 Ranges: 40.00/400.00/4000/40,000/400,000 Lux (All with Accuracy ± 3%)
High-resolution	0.01 Lux to 100 Lux
Unit	Lux or Foo-candle (Ft-cd)
Sensor	Photo diode and color correction filter spectrum meet the C.I.E. cosine correction factor standard
Selected Lighting Type	Tungsten lamp, fluorescent lamp, sodium lamp or mercury mamp
Data Output	RS-232 data output
Operating Temperature	0 °C to 50 °C

**Table 3:** Specifications of the DJI Mavic AIR 2 unmanned aerial vehicle.

Aircraft	
Max. Ascent Speed	4 m∙s^−1^ in normal mode
Max. Descent Speed	3 m∙s^−1^ in normal mode
Max. Tilt Angle	20° in normal mode and 35° in normal mode under strong wind
Operating Temperature	−10 °C to 40 °C
Satellite System	GPS + GLONASS
Camera	
Sensor	1/2“ CMOS Effective pixels: 12 MP and 48 MP
Max. Photo Resolution	48 MP 8000 × 6000 pixels

**Table 4:** Technical data of the examined Dacia Sandero vehicle.

Technical Parameter	Description
Manufacture Date	January 2015
Odometer reading	185,517 km
Body type	Hatchback
Low-beam power source	H4 55/60 W halogen bulb
High-beam power source	H4 55/60 W halogen bulb
Headlight tilt (prescribed by the manufacturer)	±1%
Light color	3200 K
Height of the headlights from the road surface (mm)	750
Distance between the headlights (mm)	1150
Type of headlight	Single-focal, paraboloid
Height, width and length of vehicle (mm)	1523, 1733, 4057
Weight of vehicle (kg)	1105
Engine, fuel	1.2 16v, petrol
Power and torque	55 kW, 107 Nm
Transmission	5-speed manual
Tire size	185/65 R15
Wheel rim size	6 J × 15

**Table 5:** Technical data of the examined Hyundai i40 vehicle.

Technical Parameter	Description
Manufacture Date	September 2012
Odometer reading	212,452 km
Body type	Station wagon (estate)
Low-beam power source	HID D1S 35 W bulb
High-beam power source	H7 55 W halogen bulb
Headlight tilt (prescribed by the manufacturer)	±1%
Light color	6000 K
Height of the headlights from the road surface (mm)	700
Distance between the headlights (mm)	1470
Type of headlight	Projection with lens
Height, width and length of vehicle (mm)	1470, 1815, 4775
Weight of vehicle (kg)	1514
Engine, fuel	1.7 CRDi, diesel
Power and torque	100 kW, 325 Nm
Transmission	6-speed automatic
Tire size	215/50 R17
Wheel rim size	7.5 J × 17

**Table 6:** Range of visibility at knee height.

Point Number	Distance from Dacia Sandero (m)	Distance from Hyundai i40 (m)
1	21.30	16.19
2	20.88	18.13
3	20.55	15.25
4	19.13	13.37
5	19.13	15.55
6	20.38	14.33
7	34.35	22.56
8	36.66	30.96
9	55.65	43.05
10	55.86	54.68
11	67.03	64.27
12	67.22	78.11

**Table 7:** Range of visibility at ankle height.

Point Number	Distance from Dacia Sandero (m)	Distance from Hyundai i40 (m)
1	43.07	37.65
2	43.19	37.63
3	45.34	37.57
4	46.67	36.01
5	48.56	35.97
6	51.49	35.96
7	54.87	39.19
8	59.81	45.74
9	63.22	58.07
10	67.72	69.20
11	75.63	80.11
12	83.32	93.44

**Table 8:** Maximum range of visibility of the vehicle.

Point Number	Distance from Dacia Sandero (m)	Distance from Hyundai i40 (m)
1	64.40	57.86
2	65.69	57.84
3	70.30	57.81
4	70.31	66.44
5	79.10	68.03
6	84.10	68.09
7	84.20	68.77
8	84.40	71.80
9	92.20	83.01
10	107.95	115.00
11	113.95	154.56
12	123.75	160.56

**Table 9:** Range of visibility at knee height on different classes of road.

Class of Road in Non-Urban Area	Dacia Sandero (m)	Hyundai i40 (m)
C 6.5 (III)	34.5	31.8
C 7.5 (II, III)	35.5	35.2
C 9.5 (I, II, III)	39.3	41.4
C 11.5 (I)	55.7	51.7
C 6.5 (III)	34.5	31.8
C 7.5 (II, III)	35.5	35.2
C 9.5 (I, II, III)	39.3	41.4
C 11.5 (I)	55.7	51.7
C 6.5 (III)	34.5	31.8
C 7.5 (II, III)	35.5	35.2
C 9.5 (I, II, III)	39.3	41.4
C 11.5 (I)	55.7	51.7

**Table 10:** Range of visibility at ankle height on different classes of road.

The Range of Visibility of a Pedestrian’s Ankle on Different Types of Highways		
Highway Type in Non-Urban Area	Dacia Sandero (m)	Hyundai i40 (m)
C 6.5 (III)	58.3	51.6
C 7.5 (II, III)	60.4	55.7
C 9.5 (I, II, III)	62.6	61.9
C 11.5 (I)	66.8	70.5

## Data Availability

The data used to support the findings of this study are available from the corresponding author upon request.
